# Supplementary material for: Mapping IgA Epitope and Cross-Reactivity between Severe Acute Respiratory Syndrome-Associated Coronavirus 2 and DENV
Source: Vaccines (Basel). 2023 Nov 24;11(12):1749. doi: 10.3390/vaccines11121749 (PMC10747746; doi:10.3390/vaccines11121749)
Supplement: Supplementary file 1 [file vaccines-11-01749-s001.zip › vaccines-2687060-supplementary.pdf]

Figure S1: Multiple sequence alignment of the six-coronaviruses spike proteins deposited in the database with a higher degree of identity (≥97%) with SARS-CoV-2. BLAST was carried out in the UniProt (Universal Protein) database of the EBI (European Bioinformatics Institute), using the P0DTC2 protein from the SARS-CoV-2 as input. HCoV-NL63 (YP_003767.1), HCoV-229E l(NP_073551.1), HCoV-HKU1 (Q14EB0.1), HCoV-OC43 (P36334.1), MERS-CoV (YP_007188579.1), SARS-CoV-2 (P0DTC2), and SARS-CoV (P59594.1).

```
              ....|....| ....|....| ....|....| ....|....| ....|....|
                      10         20         30         40         50
HCoV-NL63     --MKLFLILL VLPLASCFF- ----TCNS-N ANLS----ML QLGVPDNSST
HCoV-229E     ---------- ---------- ---------- ---------- ----------
HCoV-HKU1     -----MFLII FILPT-TLAV IGDFNCTNSF IN-----DY NKTIPRISED
HCoV-OC43     ----MFLILL ISLPT-AFAV IGDLKCTSDN IN-----DK DTGPPPISTD
MERS-CoV      MIHSVFLLMF LLTPTESYVD VGPDSVKSAC IEVDIQQTFF DKTWPRP---
SARS-CoV-2    ----MFVFLV LLPLV-S--- -------SQC VN--LTT--R TQLPPAY---
SARS-CoV      ----MFIFLL FLTLT-S--- -------GSD LD--RCTTFD DVQAPNY---

              ....|....| ....|....| ....|....| ....|....| ....|....|
                      60         70         80         90        100
HCoV-NL63     IVTGLLPTHW FCANQSTSVY SANGFFYIDV GNHRSAFALH TGYYDAN---
HCoV-229E     ---------- ---------- ---------- ---------- ----------
HCoV-HKU1     ---------- -----VVDVS LGLGTYYVLN RVYLNTTLLF TGYFPKSGAN
HCoV-OC43     ---------- -----TVDVT NGLGTYYVLD RVYLNTTLFL NGYYPTSGST
MERS-CoV      ---------- ------IDVS KADGIIYPQG RTYSNITITY QGLF-PYQGD
SARS-CoV-2    ---------- -------TNS FTRGVYYPDK VFRSSVLHST QDLFLPFFSN
SARS-CoV      ---------- -----TQHTS SMRGVYYPDE IFRSDTLYLT QDLFLPFYSN

              ....|....| ....|....| ....|....| ....|....| ....|....|
                     110        120        130        140        150
HCoV-NL63     --QYYIYVTN EIGLNASVTL KICKFSRNTT FDFLSNASSS FDCIVNLLFT
HCoV-229E     ---------- ---------- ---------- ---------- ----------
HCoV-HKU1     FRDL------ --ALKGSKYL STLW----YK PPFLSDFNNG IFSKV----K
HCoV-OC43     YRNM------ --ALKGSVLL SRLW----FK PPFLSDFING IFAKV----K
MERS-CoV      HGDMYVYS-- --AGHATGTT PQKLFVANYS QD-VKQFANG FVVRI----G
SARS-CoV-2    VT---WFH-- --AIHVSGTN GTKR----FD NP-VLPFNDG VYFAS----T
SARS-CoV      VT---GFH-- --TIN----- --HT----FG NP-VIPFKDG IYFAA----T

              ....|....| ....|....| ....|....| ....|....| ....|....|
                     160        170        180        190        200
HCoV-NL63     EQLGAPLGIT ISGETVRLHL YNVTRTFYVP AAYKLTKL-S VKCYFNYSCV
HCoV-229E     ---------- ---------- ---------- ---------- ---MFVLLVA
HCoV-HKU1     N-TKLYVNNT ---------L YSEFSTIVIG SVFVNTSY-- -------TIV
HCoV-OC43     N-TKVIKDRV ---------M YSEFPAITIG STFVNTSY-- -------SVV
MERS-CoV      A-AANSTGTV IISPSTSATI RKIYPAFMLG SSVGNFSDGK MGRFFNHTLV
SARS-CoV-2    E--------- ---------K SNIIRGWIFG TTLDSKTQ-- -------SLL
SARS-CoV      E--------- ---------K SNVVRGWVFG STMNNKSQ-- -------SVI

              ....|....| ....|....| ....|....| ....|....| ....|....|
                     210        220        230        240        250
HCoV-NL63     FSVVNATVTV NVTTHNG--- --RVVNYTVC DDCNGYTDNI FSVQQDGRIP
HCoV-229E     YA---LLHIA GCQTTNG--- --LNTSYSVC NGCVGYSENV FAVESGGYIP
HCoV-HKU1     V----QPH-- ---------- -NGILEITAC Q--------- ----------
HCoV-OC43     V----QPRTI NST-QDGDNK LQGLLEVSVC Q--------- ----------
MERS-CoV      L----LPD-- ------G--- --CGTLLRAF ---------- ----------
SARS-CoV-2    I----VNN-- ------A--- --TNVVIKVC E--------- ----------
SARS-CoV      I----INN-- ------S--- --TNVVIRAC N--------- ----------
```

```
             ....|....| ....|....| ....|....| ....|....| ....|....|
                    260        270        280        290        300
HCoV-NL63    NGFPFNNWFL LTNGSTLVDG VSRLYQPLRL TCLWPVPGLK SSTGFVYF--
HCoV-229E    SDFAFNNWFL LTNTSSVVDG VVRSFQPLLL NCLWSVSGLR FTTGFVYF--
HCoV-HKU1    ---------- ---------- --------YT MCEYPHTVCK S-KGSIRN--
HCoV-OC43    ---------- ---------- --------YN MCEYPQTICH PNLGNHRK--
MERS-CoV     ---------- ---------- ---------Y CILEPRSGNH CPAGNSYTSF
SARS-CoV-2   ---------- ---------- --------FQ FCNDPFLGVY YHKNN-----
SARS-CoV     ---------- ---------- --------FE LCDNPFFAVS KPMGT-----

             ....|....| ....|....| ....|....| ....|....| ....|....|
                    310        320        330        340        350
HCoV-NL63    ---NATGSDV NCNGYQHNSV VD-----VMR YNLNFSANSL ---DNLKSG-
HCoV-229E    ---NGTGR-G DCKGFSSDVL SD-----VIR YNLNFEE--- ----NLRRG-
HCoV-HKU1    ---------- ------ESWH IDSSE-PLCL FKKNFTYNVS A------DWL
HCoV-OC43    ---------- ------ELWH LDTGV-VSCL YKRNFTYDVN A------DYL
MERS-CoV     ATYHTPATDC SDGNYNRNAS LNSFKEYFNL RNCTFMYTYN ITEDEILEWF
SARS-CoV-2   ---------- ------KSWM ESEFRVYSSA NNCTFEYVSQ PFLMDLEGKQ
SARS-CoV     ---------- ---------- QTHTMIFDNA FNCTFEYISD AFSLDVSEKS

             ....|....| ....|....| ....|....| ....|....| ....|....|
                    360        370        380        390        400
HCoV-NL63    -------VIV FKTLQYDVLF YCSNSSSGV- ---------- -LDTTIPFGP
HCoV-229E    -------TIL FKTSYGVVVF YCTNNTLVS- ---------- -GDAHIPFGT
HCoV-HKU1    -------YFH FYQERGVFYA YYADV--G-- ------MP-T TFLFSLYLGT
HCoV-OC43    -------YFH FYQEGGTFYA YFTDT--G-- ------VV-T KFLFNVYLGM
MERS-CoV     GITQTAQG-V HLFSSRYVDL YGGN------ ---------M FQFATLPVYD
SARS-CoV-2   GNFKNLREFV FKNIDGYFKI YSKHTPINLV RDLPQGFSAL EPLVDLPIGI
SARS-CoV     GNFKHLREFV FKNKDGFLYV YKGYQPIDVV RDLPSGFNTL KPIFKLPLGI

             ....|....| ....|....| ....|....| ....|....| ....|....|
                    410        420        430        440        450
HCoV-NL63    SSQPYYCFIN STINTTHVST FVGILPPTVR EIVVARTGQF YINGFKYFDL
HCoV-229E    VLGNFYCFVN TTIGNETTSA FVGALPKTVR EFVISRTGHF YINGYRYFTL
HCoV-HKU1    ILSHYYVM-- ---------- -----PLTCK ---------- ----------
HCoV-OC43    ALSHYYVM-- ---------- -----PLTCN ---------- ----------
MERS-CoV     TIKYYSII-- ---------- -----PHSIR ---------- --------SI
SARS-CoV-2   NITRFQTL-- ---------- -----LALHR SYL------- --------TP
SARS-CoV     NITNFRAI-- ---------- -----LTAFS ---------- ----------

             ....|....| ....|....| ....|....| ....|....| ....|....|
                    460        470        480        490        500
HCoV-NL63    GFIEAVNFNV TTASATDFWT VAFATFVDVL VNVSATNIQN LLYCD-SPFE
HCoV-229E    GNVEAVNFNV TTAETTDFCT VALASYADVL VNVSQTSIAN IIYCN-SVIN
HCoV-HKU1    -A----ISSN TDNETLEYWV TPLSRRQYLL NFDEHGVITN AVDCSSSFLS
HCoV-OC43    -S-------- --KLTLEYWV TPLTSRQYLL AFNQDGIIFN AEDCMSDFMS
MERS-CoV     QS----DRKA W---AAFYV YKLQPLTFLL DFSVDGYIRR AIDCGFNDLS
SARS-CoV-2   GD----SSSG WTAGAAAYYV GYLQPRTFLL KYNENGTITD AVDCALDPLS
SARS-CoV     -P----AQDI WGTSAAAYFV GYLKPTTFML KYDENGTITD AVDCSQNPLA

             ....|....| ....|....| ....|....| ....|....| ....|....|
                    510        520        530        540        550
HCoV-NL63    KLQCEHLQFG LQDGFYSANF LDDNVLPETY VALPIY---- ----------
HCoV-229E    RLRCDQLSFD VPDGFYSTSP IQSVELPVSI VSLPVY---- ----------
HCoV-HKU1    EIQCKTQSFA PNTGVYDLSG FTVKPVATVY RRIPNLPDCD IDNWLNNVSV
HCoV-OC43    EIKCKTQSIA PPTGVYELNG YTVQPIADVY RRKPNLPNCN IEAWLNDKSV
MERS-CoV     QLHCSYESFD VESGVYSVSS FEAKPSGSVV EQAEG-VECD FSPLLSG-TP
```

```
SARS-CoV-2    ETKCTLKSFT VEKGIYQTSN FRVQPTESIV RFPNITNLCP FGEVFNATRF
SARS-CoV      ELKCSVKSFE IDKGIYQTSN FRVVPSGDVV RFPNITNLCP FGEVFNATKF

              ....|....| ....|....| ....|....| ....|....| ....|....|
                     560        570        580        590        600
HCoV-NL63     ------YQHT DINFT----- ---------- -----ATASF GGSCYVCKPH
HCoV-229E     ------HKHT FIVLYV---- ---------- ---DFKPQSG GGKCFNCYPA
HCoV-HKU1     PSPLNWERRI FSNCNFNLST LLRLVHVDSF SCNNLDKSKI FGSCFNSI--
HCoV-OC43     PSPLNWERKT FSNCNFNMSS LMSFIQADSF TCNNIDAAKI YGMCFSSI--
MERS-CoV      PQVYNFKRLV FTNCNYNLTK LLSLFSVNDF TCSQISPAAI ASNCYSSL--
SARS-CoV-2    ASVYAWNRKR ISNCVADYSV LYNSASFSTF KCYGVSPTKL NDLCFTNV--
SARS-CoV      PSVYAWERKK ISNCVADYSV LYNSTFFSTF KCYGVSATKL NDLCFSNV--

              ....|....| ....|....| ....|....| ....|....| ....|....|
                     610        620        630        640        650
HCoV-NL63     QVNISLNG-- -----NTSVC VRTSHFSIRY IYNRVKSGSP GDSSWHIYLK
HCoV-229E     GVNITLANFN ET---KGPLC VDTSHFTTKY VAVYA----- NVGRWSASIN
HCoV-HKU1     ----TVDKFA IPNRRRDDLQ LGSSGFLQSS NYKIDISSSS CQLYYSLPLV
HCoV-OC43     ----TIDKFA IPNGRKVDLQ LGNLGYLQSF NYRIDTTATS CQLYYNLPAA
MERS-CoV      ----ILDYFS YPLSMKSDLS VSSAGPISQF NYKQSFSNPT CLILATVPHN
SARS-CoV-2    ----YADSFV IRGDEVRQIA PGQTGKIADY NYKLPDDFTG CVIAWNSNNL
SARS-CoV      ----YADSFV VKGDDVRQIA PGQTGVIADY NYKLPDDFMG CVLAWNTRNI

              ....|....| ....|....| ....|....| ....|....| ....|....|
                     660        670        680        690        700
HCoV-NL63     --SGTCPF-- -------SFS KLNNFQ---- --------KF KTICFSTVEV
HCoV-229E     --TGNCPF-- -------SFG KVNNFV---- --------KF GSVCFSLKDI
HCoV-HKU1     NVTINN-FNP SSWNRRYGFG SFN------- --VSSYDVVY SDHCFSVNSD
HCoV-OC43     NVSVSR-FNP STWNKRFGFI EDSVFKPRPA GVLTNHDVVY AQHCFKAPKN
MERS-CoV      LTTITKPLKY SYINKCSRFL SD-----DRT E--------V PQLVNA--NQ
SARS-CoV-2    DSKVGGNYNY LYRLFRKSNL KP-----FER D--------I STEIYQ--AG
SARS-CoV      DATSTGNYNY KYRYLRHGKL RP-----FER D--------I SNVPFS--PD

              ....|....| ....|....| ....|....| ....|....| ....|....|
                     710        720        730        740        750
HCoV-NL63     P--------- GSCNF----- ---------- ---------- ----------
HCoV-229E     P--------- GGCAM----- ---------- ---------- ----------
HCoV-HKU1     FCPCADPSVV NSCVKSKP-- --LSAICPAG TKYRHCDLDT TLYVNNWCRC
HCoV-OC43     FCPCKLN--- GSCVGSGPGK NNGIGTCPAG TNYLTCD--- ---------N
MERS-CoV      YSPCVSIVP- ST-VW----- -------EDG DYYRK----- ----------
SARS-CoV-2    STPCNGVEG- FNCYF----- ---------- ---------- ----------
SARS-CoV      GKPCTP-PA- LNCYW----- ---------- ---------- ----------

              ....|....| ....|....| ....|....| ....|....| ....|....|
                     760        770        780        790        800
HCoV-NL63     -----PLEAT WHYT------ ---------- ---------- -----SYTIV
HCoV-229E     -----PIVAN WAYS------ ---------- ---------- -----KYYTI
HCoV-HKU1     SCLPDPISTY SPNTCPQKKV VVGIGEHCPG LGINEEKCGT QLNHSSCSCS
HCoV-OC43     LCTPDPITFT GTYKCPQTKS LVGIGEHCSG LAVKSDYCGG ----NSCTCR
MERS-CoV      --QLSPLEGG GWLV------ ---------- ---------- ---ASGSTVA
SARS-CoV-2    -----PLQSY GFQP------ ---------- ---------- ---TNGVGYQ
SARS-CoV      -----PLNDY GFYT------ ---------- ---------- ---TTGIGYQ

              ....|....| ....|....| ....|....| ....|....| ....|....|
                     810        820        830        840        850
HCoV-NL63     GALYVTWSEG NSITGV---- ---------- PYPV-----S GIREFSNLVL
HCoV-229E     GSLYVSWSDG DGITGV---- ---------- PQPV-----E GVSSFMNVTL
```

```
HCoV-HKU1    PDAFLGWSFD SCISNNRCNI FSNFIFNGIN SGTTCSN--D LLYSNTEVST
HCoV-OC43    PQAFLGWSAD SCLQGDKCNI FANFILHDVN SGLTCST--D LQKANTDIIL
MERS-CoV     MTEQLQMGFG IT-------- ----VQYGTD TNSVCPKLEF ANDTKIASQL
SARS-CoV-2   PYRVVVLSFE LL-------- --------HA PATVCGP--- --KKSTNLVK
SARS-CoV     PYRVVVLSFE LL-------- --------NA PATVCGP--- --KLSTDLIK

             ....|....| ....|....| ....|....| ....|....| ....|....|
                    860        870        880        890        900
HCoV-NL63    NNCTKYNIYD YVGTGIIRSS NQSLAGGIT- -YVSNSGNLL G-FKNVSTGN
HCoV-229E    DKCTKYNIYD VSGVGVIRVS NDTFLNGIT- -YTSTSGNLL G-FKDVTKGT
HCoV-HKU1    GVCVNYDLYG ITGQGIFKEV SAAYYNNWQN LLYDSNGNII G-FKDFLTNK
HCoV-OC43    GVCVNYDLYG ILGQGIFVEV NATYYNSWQN LLYDSNGNLY G-FRDYIINR
MERS-CoV     GNCVEYSLYG VSGRGVFQNC TAVG-VRQQR FVYDAYQNLV GYYSD--DGN
SARS-CoV-2   NKCVNFNFNG LTGTGVLTES NKKF-LPFQQ FGRDIADTT- DAVRDPQTLE
SARS-CoV     NQCVNFNFNG LTGTGVLTPS SKRF-QPFQQ FGRDVSDFT- DSVRDPKTSE

             ....|....| ....|....| ....|....| ....|....| ....|....|
                    910        920        930        940        950
HCoV-NL63    IFIVTPCNQP DQVAVYQQ-- ---------- SIIGAMTAVN ESRYGLQ---
HCoV-229E    IYSITPCNPP DQLVVYQQ-- ---------- AVVGAMLSEN FTSYGFS---
HCoV-HKU1    TYTILPCYSG RVSAAFY--Q NSSSPALLYR NLKCSYVLNN IS--FIS---
HCoV-OC43    TFMIRSCYSG RVSAAFH--A NSSEPALLFR NIKCNYVFNN SLTRQLQ---
MERS-CoV     YYCLRACVSV PVSVIYD--K ETKTHATLFG SVACEHISST MSQYSRSTRS
SARS-CoV-2   ILDITPCSFG GVSVITPGTN TSNQVAVLYQ DVNCTEVPVA IHADQLT--P
SARS-CoV     ILDISPCSFG GVSVITPGTN ASSEVAVLYQ DVNCTDVSTA IHADQLT--P

             ....|....| ....|....| ....|....| ....|....| ....|....|
                    960        970        980        990       1000
HCoV-NL63    --NLLQLPNF YYVSNGG--- --NNCT---- TAVMTYSNFG ICADGSLIP-
HCoV-229E    --NVVELPKF FYASNGT--- --YNCT---- DAVLTYSSFG VCADGSIIA-
HCoV-HKU1    -------QPF YFDSYLGCVL NAVNLTSYSV SSCDLRMGSG FCIDYALPSS
HCoV-OC43    -------PIN YFDSYLGCVV NAYNSTAISV QTCDLTVGSG YCVDYSK--N
MERS-CoV     MLKRRDSTYG PLQTPVGCVL GLVNSS-LFV EDCKLPLGQS LCALPDTPST
SARS-CoV-2   TWRVYSTGSN VFQTRAGCLI GAEHVN--NS YECDIPIGAG ICASYQTQT-
SARS-CoV     AWRIYSTGNN VFQTQAGCLI GAEHVD--TS YECDIPIGAG ICASYHTVS-

             ....|....| ....|....| ....|....| ....|....| ....|....|
                   1010       1020       1030       1040       1050
HCoV-NL63    VRPR---NSS DNGIS----- ---------- -AIITANLSI PSNWTTSVQV
HCoV-229E    VQPR---NVS YDSVS----- ---------- -AIVTANLSI PSNWTTSVQV
HCoV-HKU1    RRKRRGISSP YRFVT-FEPF NVSFVNDSVE TVGGLFEIQI PTNFTIAGHE
HCoV-OC43    RRSRGAITTG YRFTN-FEPF TVNSVNDSLE PVGGLYEIQI PSEFTIGNMV
MERS-CoV     LTPRSVRSVP GEMRLASIAF NHPIQ-V-DQ LNSSYFKLSI PTNFSFGVTQ
SARS-CoV-2   NSPRRARSVA SQSI---IAY TMSLG-AENS VAYSNNSIAI PTNFTISVTT
SARS-CoV     L----LRSTS QKSI---VAY TMSLG-ADSS IAYSNNTIAI PTNFSISITT

             ....|....| ....|....| ....|....| ....|....| ....|....|
                   1060       1070       1080       1090       1100
HCoV-NL63    EYLQITSTPI VVDCATYVCN GNPRCKNLLK QYTSACKTIE DALRLSAHLE
HCoV-229E    EYLQITSTPI VVDCSTYVCN GNVRCVELLK QYTSACKTIE DALRNSARLE
HCoV-HKU1    EFIQTSSPKV TIDCSAFVCS NYAACHDLLS EYGTFCDNIN SILNEVNDLL
HCoV-OC43    EFIQTSSPKV TIDCAAFVCG DYAACKSQLV EYGSFCDNIN AILTEVNELL
MERS-CoV     EYIQTTIQKV TVDCKQYVCN GFQKCEQLLR EYGQFCSKIN QALHGANLRQ
SARS-CoV-2   EILPVSMTKT SVDCTMYICG DSTECSNLLL QYGSFCTQLN RALTGIAVEQ
SARS-CoV     EVMPVSMAKT SVDCNMYICG DSTECANLLL QYGSFCTQLN RALSGIAAEQ
```

```
             ....|....| ....|....| ....|....| ....|....| ....|....|
                   1110       1120       1130       1140       1150
HCoV-NL63    TNDVSSMLTF DSNAFSLAN- ----VTSFGD -YNLSSVLPQ RNIRSSRIAG
HCoV-229E    SADVSEMLTF DKKAFTLAN- ----VSSFGD -YNLSSVIPS LPTSGSRVAG
HCoV-HKU1    DITQLQVANA LMQGVTLSSN LNTNLHSDVD NIDFKSLLGC LGSQCGS-SS
HCoV-OC43    DTTQLQVANS LMNGVTLSTK LKDGVNFNVD DINFSPVLGC LGSECSKASS
MERS-CoV     DDSVRNLFAS VKSSQSSPI- ----IPGFGG DFNLTLLEP- VSISTGSRSA
SARS-CoV-2   DKNTQEVFAQ VKQIYKTPP- ----IKDFGG F-NFSQILP- DP---SKPSK
SARS-CoV     DRNTREVFAQ VKQMYKTPT- ----LKYFGG F-NFSQILP- DP---LKPTK

             ....|....| ....|....| ....|....| ....|....| ....|....|
                   1160       1170       1180       1190       1200
HCoV-NL63    RSALEDLLFS KVVTSGLGTV DVDYKSCT-- KGLSIADLAC AQYYNGIMVL
HCoV-229E    RSAIEDILFS KLVTSGLGTV DADYKKCT-- KGLSIADLAC AQYYNGIMVL
HCoV-HKU1    RSLLEDLLFN KVKLSDVGFV E-AYNNCT-- GGSEIRDLLC VQSFNGIKVL
HCoV-OC43    RSAIEDLLFD KVKLSDVGFV E-AYNNCT-- GGAEIRDLIC VQSYKGIKVL
MERS-CoV     RSAIEDLLFD KVTIADPGYM Q-GYDDCMQQ GPASARDLIC AQYVAGYKVL
SARS-CoV-2   RSFIEDLLFN KVTLADAGFI K-QYGDCL-- GDIAARDLIC AQKFNGLTVL
SARS-CoV     RSFIEDLLFN KVTLADAGFM K-QYGECL-- GDINARDLIC AQKFNGLTVL

             ....|....| ....|....| ....|....| ....|....| ....|....|
                   1210       1220       1230       1240       1250
HCoV-NL63    PGVADAERMA MYTGSLIGGM VLGGLTSA-- --AAIPFSLA LQARLNYVAL
HCoV-229E    PGVADAERMA MYTGSLIGGI ALGGLTSA-- --VSIPFSLA IQARLNYVAL
HCoV-HKU1    PPILSETQIS GYTTAATVAA MFPPWSAA-- --AGVPFSLN VQYRINGLGV
HCoV-OC43    PPLLSENQIS GYTLAATSAS LFPPWTAA-- --AGVPFYLN VQYRINGLGV
MERS-CoV     PPLMDVNMEA AYTSSLLGSI AGVGWTAGLS SFAAIPFAQS IFYRLNGVGI
SARS-CoV-2   PPLLTDEMIA QYTSALLAGT ITSGWTFGAG AALQIPFAMQ MAYRFNGIGV
SARS-CoV     PPLLTDDMIA AYTAALVSGT ATAGWTFGAG AALQIPFAMQ MAYRFNGIGV

             ....|....| ....|....| ....|....| ....|....| ....|....|
                   1260       1270       1280       1290       1300
HCoV-NL63    QTDVLQENQK ILAASFNKAI NNIVASFSSV NDAITQTAEA IHTVTIALNK
HCoV-229E    QTDVLQENQK ILAASFNKAM TNIVDAFTGV NDAITQTSQA LQTVATALNK
HCoV-HKU1    TMDVLNKNQK LIANAFNKAL LSIQNGFTAT N--------- -----SALAK
HCoV-OC43    TMDVLSQNQK LIANAFNNAL YAIQEGFDAT N--------- -----SALVK
MERS-CoV     TQQVLSENQK LIANKFNQAL GAMQTGFTTT N--------- -----EAFHK
SARS-CoV-2   TQNVLYENQK LIANQFNSAI GKIQDSLSST A--------- -----SALGK
SARS-CoV     TQNVLYENQK QIANQFNKAI SQIQESLTTT S--------- -----TALGK

             ....|....| ....|....| ....|....| ....|....| ....|....|
                   1310       1320       1330       1340       1350
HCoV-NL63    IQDVVNQQGS ALNHLTSQLR HNFQAISNSI QAIYDRLDSI QADQQVDRLI
HCoV-229E    IQDVVNQQGN SLNHLTSQLR QNFQAISSSI QAIYDRLDTI QADQQVDRLI
HCoV-HKU1    IQSVVNANAQ ALNSLLQQLF NKFGAISSSL QEILSRLDNL EAQVQIDRLI
HCoV-OC43    IQAVVNANAE ALNNLLQQLS NRFGAISASL QEILSRLDAL EAEAQIDRLI
MERS-CoV     VQDAVNNNAQ ALSKLASELS NTFGAISASI GDIIQRLDVL EQDAQIDRLI
SARS-CoV-2   LQDVVNQNAQ ALNTLVKQLS SNFGAISSVL NDILSRLDKV EAEVQIDRLI
SARS-CoV     LQDVVNQNAQ ALNTLVKQLS SNFGAISSVL NDILSRLDKV EAEVQIDRLI

             ....|....| ....|....| ....|....| ....|....| ....|....|
                   1360       1370       1380       1390       1400
HCoV-NL63    TGRLAALNAF VSQVLNKYTE VRGSRRLAQQ KINECVKSQS NRYGFCGNGT
HCoV-229E    TGRLAALNVF VSHTLTKYTE VRASRQLAQQ KVNECVKSQS KRYGFCGNGT
HCoV-HKU1    NGRLTALNAY VSQQLSDITL IKAGASRAIE KVNECVKSQS PRINFCGNGN
HCoV-OC43    NGRLTALNAY VSQQLSDSTL VKFSAAQAME KVNECVKSQS SRINFCGNGN
MERS-CoV     NGRLTTLNAF VAQQLVRSES AALSAQLAKD KVNECVKAQS KRSGFCGQGT
```

```
SARS-CoV-2    TGRLQSLQTY VTQQLIRAAE IRASANLAAT KMSECVLGQS KRVDFCGKGY
SARS-CoV      TGRLQSLQTY VTQQLIRAAE IRASANLAAT KMSECVLGQS KRVDFCGKGY

              ....|....| ....|....| ....|....| ....|....| ....|....|
                    1410       1420       1430       1440       1450
HCoV-NL63     HIFSIVNSAP DGLLFLHTVL LPTDYKNVKA WSGICVDGIY --GYVLRQPN
HCoV-229E     HIFSIVNAAP EGLVFLHTVL LPTQYKDVEA WSGLCVDGTN --GYVLRQPN
HCoV-HKU1     HILSLVQNAP YGLLFIHFSY KPTSFKTVLV SPGLCLSGDR --GIAPKQGY
HCoV-OC43     HIISLVQNAP YGLYFIHFSY VPTKYVTARV SPGLCIAGDR --GIAPKSGY
MERS-CoV      HIVSFVVNAP NGLYFMHVGY YPSNHIEVVS AYGLCDAANP TNCIAPVNGY
SARS-CoV-2    HLMSFPQSAP HGVVFLHVTY VPAQEKNFTT APAICHDGKA ---HFPREGV
SARS-CoV      HLMSFPQAAP HGVVFLHVTY VPSQERNFTT APAICHEGKA ---YFPREGV

              ....|....| ....|....| ....|....| ....|....| ....|....|
                    1460       1470       1480       1490       1500
HCoV-NL63     LVLY---SDN GVFRVTSRVM FQPRLPVLSD FVQIYNCNVT FVNISRVEL-
HCoV-229E     LALY---KEG NYYRITSRIM FEPRIPTMAD FVQIENCNVT FVNISRSEL-
HCoV-HKU1     FIKQ-----N DSWMFTGSSY YYPEPISDKN VVFMNSCSVN FTKAPFIYL-
HCoV-OC43     FVNV-----N NTWMYTGSGY YYPEPITENN VVVMSTCAVN YTKAPYVML-
MERS-CoV      FIKTNNTRIV DEWSYTGSSF YAPEPITSLN TKYVAPQVTY QN-ISTNLPP
SARS-CoV-2    FVSN-----G THWFVTQRNF YEPQIITTDN TFVSGNCDVV IGIVNNTVYD
SARS-CoV      FVFN-----G TSWFITQRNF FSPQIITTDN TFVSGNCDVV IGIINNTVYD

              ....|....| ....|....| ....|....| ....|....| ....|....|
                    1510       1520       1530       1540       1550
HCoV-NL63     -HTVIPDYVD VNKTLQEFAQ NLPKYVKPNF -DLTPFNLTY LNLSSELKQL
HCoV-229E     -QTIVPEYID VNKTLQELSY KLPNYTVPDL -VVEQYNQTI LNLTSEISTL
HCoV-HKU1     -NNSIPNLSD FEAEFSLWFK NHTSI-APNL TFNSHINATF LDLYY-----
HCoV-OC43     -NTSIPNLPD FKEELDQWFK NQTSV-APDL SLD-YINVTF LDLQV-----
MERS-CoV      PLLGNSTGID FQDELDEFFK NVSTS-IPNF GSLTQINTTL LDLTY-----
SARS-CoV-2    PLQ--PELDS FKEELDKYFK NHTSP-DVDL GDISGINASV VNIQK-----
SARS-CoV      PLQ--PELDS FKEELDKYFK NHTSP-DVDL GDISGINASV VNIQK-----

              ....|....| ....|....| ....|....| ....|....| ....|....|
                    1560       1570       1580       1590       1600
HCoV-NL63     EAKTASLFQT TVELQGLIDQ INSTYVDLKL LNRFENYIKW PWWVWLIISV
HCoV-229E     ENKSAELNYT VQKLQTLIDN INSTLVDLKW LNRVETYIKW PWWVWLCISV
HCoV-HKU1     ---------E MNVIQESIKS LNSSFINLKE IGTYEMYVKW PWYIWLLIVI
HCoV-OC43     ---------E MNRLQEAIKV LNQSYINLKD IGTYEYYVKW PWYVWLLICL
MERS-CoV      ---------E MLSLQQVVKA LNESYIDLKE LGNYTYYNKW PWYIWLGFIA
SARS-CoV-2    ---------E IDRLNEVAKN LNESLIDLQE LGKYEQYIKW PWYIWLGFIA
SARS-CoV      ---------E IDRLNEVAKN LNESLIDLQE LGKYEQYIKW PWYVWLGFIA

              ....|....| ....|....| ....|....| ....|....| ....|....|
                    1610       1620       1630       1640       1650
HCoV-NL63     VFVVLLSLLV FCCLSTGCCG CCNCLTSSMR GCCDCGSTKL PYYEFEKVHV
HCoV-229E     VLIFVVSMLL LCCCSTGCCG FFSCFASSIR GCCE--STKL PYYDVEKIHI
HCoV-HKU1     LFIIFLMILF FICCCTGCGS ACF------- S--KCHNCCD EYGGHNDFVI
HCoV-OC43     AGVAMLVLLF FICCCTGCGT SCF------- K--KCGGCCD DYTGYQELVI
MERS-CoV      GLVALALCVF FILCCTGCGT NCM------- GKLKCNRCCD RYEEYDLEPH
SARS-CoV-2    GLIAIVMVTI MLCCMTSCCS CLK------- GCCSCGSCCK F-DEDDSEPV
SARS-CoV      GLIAIVMVTI LLCCMTSCCS CLK------- GACSCGSCCK F-DEDDSEPV
```

HCoV-NL63	Q--------
HCoV-229E	Q--------
HCoV-HKU1	K-ASHDD--
HCoV-OC43	K-TSHDD--
MERS-CoV	KVHVH----
SARS-CoV-2	LKGVKLHYT
SARS-CoV	LKGVKLHYT

Figure S2: Analysis of pre-pandemic sera from patients with DENV (n = 24) and sera from hospitalized patients with SARS-CoV-2 (first wave) using a commercial enzyme-linked immunosorbent assay for IgA-SARS-CoV-2. The methodology described by the manufacturer (Serion Immunomat, Würzburg, Germany).

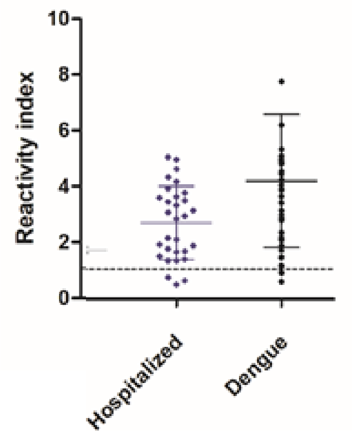

Table S1. Clinical characteristics of COVID-19 patients sera used in the study. [1] Mild Illness: Individuals presenting with various signs and symptoms of COVID-19, such as cough, fever, sore throat, malaise, headache, myalgias, nausea, vomiting, gastrointestinal symptoms, and loss of taste and smell. However, they do not exhibit shortness of breath, dyspnea, or abnormal chest imaging. [2] Moderate Illness: Individuals who display evidence of lower respiratory disease during clinical assessment or imaging and maintain an oxygen saturation measured by pulse oximetry (SpO2) of ≥94% while breathing room air at sea level. [3] Severe Illness: Individuals with a SpO2 measurement of <94% on room air at sea level, a ratio of arterial partial pressure of oxygen to fraction of inspired oxygen (PaO2/FiO2) below 300 mm Hg, a respiratory rate exceeding 30 breaths per minute, or lung infiltrates covering more than 50% of their lung field.

	**COVID (n= 33)**	**Mild[1] (n= 5)**	**Mod[2] (n=12)**	**Severe[3] (n=16)**	**P-value**
Median Age (IQR)	(27–48)	(26–39)	(27–47)	(41–58)	<0.001
Age group, n (%)	100	15.16	36.36	48.48	<0.001
• < 20	4 (50)	2 (25)	1 (12.5)	1 (12.5)	
• 20–39	17 (50)	1 (2.94)	10 (29,41)	6 (17.64)	
• 40–59	12 (50)	2 (8.33)	1 (4.16)	9 (37.50)	
Male, n (%)	19 (50)	3 (7.89)	7 (18.42)	9 (23.68)	<0.001

[1]Mild Illness: Individuals presenting with various signs and symptoms of COVID-19, such as cough, fever, sore throat, malaise, headache, myalgias, nausea, vomiting, gastrointestinal symptoms, and loss of taste and smell. However, they do not exhibit shortness of breath, dyspnea, or abnormal chest imaging. 2Moderate Illness: Individuals who display evidence of lower respiratory disease during clinical assessment or imaging and maintain an oxygen saturation measured by pulse oximetry (SpO2) of ≥94% while breathing room air at sea level. [3]Severe Illness: Individuals with a SpO2 measurement of <94% on room air at sea level, a ratio of arterial partial pressure of oxygen to fraction of inspired oxygen (PaO2/FiO2) below 300 mm Hg, a respiratory rate exceeding 30 breaths per minute, or lung infiltrates covering more than 50% of their lung field.

Table S2: List of synthesized peptides covering the entire sequence of spike protein (P0DTC2, Uniprot database) from SARS-CoV-2. Positive controls (F3, F4, F5, F11, F12, F13) and negative controls (F9, F17). The overlapping of positive peptides defined by the epitopes is labeled in red.

Spot	Peptide Sequence	Spot	Peptide Sequence	Spot	Peptide Sequence	Spot	Peptide Sequence
A1	IHLVNNESSEVIVHK	C17	LSETKCTLKSFTVEK	F9	NCTEVPVAIHADQLT	I1	DSLSSTASALGKLQD
A2	GYPKDGNAFNNLDRI	C18	CTLKSFTVEKGIYQT	F10	PVAIHADQLTPTWRV	I2	TASALGKLQDVVNQN
A3	KEVPALTAVETGATN	C19	FTVEKGIYQTSNFRV	F11	ADQLTPTWRVYSTGS	I3	GKLQDVVNQNAQALN
A4	YPYDVPDYAGYPYDV	C20	GIYQTSNFRVQPTES	F12	PTWRVYSTGSNVFQT	I4	VVNQNAQALNTLVKQ
A5		C21	SNFRVQPTESIVRFP	F13	YSTGSNVFQTRAGCL	I5	AQALNTLVKQLSSNF
A6	MFVFLVLLPLVSSQC	C22	QPTESIVRFPNITNL	F14	NVFQTRAGCLIGAEH	I6	TLVKQLSSNFGAISS
A7	VLLPLVSSQCVNLTT	C23	IVRFPNITNLCPFGE	F15	RAGCLIGAEHVNNSY	I7	LSSNFGAISSVLNDI
A8	VSSQCVNLTTRTQLP	C24	NITNLCPFGEVFNAT	F16	IGAEHVNNSYECDIP	I8	GAISSVLNDILSRLD
A9	VNLTTRTQLPPAYTN	D1	CPFGEVFNATRFASV	F17	VNNSYECDIPIGAGI	I9	VLNDILSRLDKVEAE
A10	RTQLPPAYTNSFTRG	D2	VFNATRFASVYAWNR	F18	ECDIPIGAGICASYQ	I10	LSRLDKVEAEVQIDR
A11	PAYTNSFTRGVYYPD	D3	RFASVYAWNRKRISN	F19	IGAGICASYQTQTNS	I11	KVEAEVQIDRLITGR
A12	SFTRGVYYPDKVFRS	D4	YAWNRKRISNCVADY	F20	CASYQTQTNSPRRAR	I12	VQIDRLITGRLQSLQ
A13	VYYPDKVFRSSVLHS	D5	KRISNCVADYSVLYN	F21	TQTNSPRRARSVASQ	I13	LITGRLQSLQTYVTQ
A14	KVFRSSVLHSTQDLF	D6	CVADYSVLYNSASFS	F22	PRRARSVASQSIIAY	I14	LQSLQTYVTQQLIRA
A15	SVLHSTQDLFLPFFS	D7	SVLYNSASFSTFKCY	F23	SVASQSIIAYTMSLG	I15	TYVTQQLIRAAEIRA
A16	TQDLFLPFFSNVTWF	D8	SASFSTFKCYGVSPT	F24	SIIAYTMSLGAENSV	I16	QLIRAAEIRASANLA
A17	LPFFSNVTWFHAIHV	D9	TFKCYGVSPTKLNDL	G1	TMSLGAENSVAYSNN	I17	AEIRASANLAATKMS
A18	NVTWFHAIHVSGTNG	D10	GVSPTKLNDLCFTNV	G2	AENSVAYSNNSIAIP	I18	SANLAATKMSECVLG
A19	HAIHVSGTNGTKRFD	D11	KLNDLCFTNVYADSF	G3	AYSNNSIAIPTNFTI	I19	ATKMSECVLGQSKRV
A20	SGTNGTKRFDNPVLP	D12	CFTNVYADSFVIRGD	G4	SIAIPTNFTISVTTE	I20	ECVLGQSKRVDFCGK
A21	TKRFDNPVLPFNDGV	D13	YADSFVIRGDEVRQI	G5	TNFTISVTTEILPVS	I21	QSKRVDFCGKGYHLM
A22	NPVLPFNDGVYFAST	D14	VIRGDEVRQIAPGQT	G6	SVTTEILPVSMTKTS	I22	DFCGKGYHLMSFPQS
A23	FNDGVYFASTEKSNI	D15	EVRQIAPGQTGKIAD	G7	ILPVSMTKTSVDCTM	I23	GYHLMSFPQSAPHGV
A24	YFASTEKSNIIRGWI	D16	APGQTGKIADYNYKL	G8	MTKTSVDCTMYICGD	I24	SFPQSAPHGVVFLHV
B1	EKSNIIRGWIFGTTL	D17	GKIADYNYKLPDDFT	G9	VDCTMYICGDSTECS	J1	APHGVVFLHVTYVPA

B2	IRGWIFGTTLDSKTQ	D18	YNYKLPDDFTGCVIA	G10	YICGDSTECSNLLLQ	J2	VFLHVTYVPAQEKNF
B3	FGTTLDSKTQSLLIV	D19	PDDFTGCVIAWNSNN	G11	STECSNLLLQYGSFC	J3	TYVPAQEKNFTTAPA
B4	DSKTQSLLIVNNATN	D20	GCVIAWNSNNLDSKV	G12	NLLLQYGSFCTQLNR	J4	QEKNFTTAPAICHDG
B5	SLLIVNNATNVVIKV	D21	WNSNNLDSKVGGNYN	G13	YGSFCTQLNRALTGI	J5	TTAPAICHDGKAHFP
B6	NNATNVVIKVCEFQF	D22	LDSKVGGNYNYLYRL	G14	TQLNRALTGIAVEQD	J6	ICHDGKAHFPREGVF
B7	VVIKVCEFQFCNDPF	D23	GGNYNYLYRLFRKSN	G15	ALTGIAVEQDKNTQE	J7	KAHFPREGVFVSNGT
B8	CEFQFCNDPFLGVYY	D24	YLYRLFRKSNLKPFE	G16	AVEQDKNTQEVFAQV	J8	REGVFVSNGTHWFVT
B9	CNDPFLGVYYHKNNK	E1	FRKSNLKPFERDIST	G17	KNTQEVFAQVKQIYK	J9	VSNGTHWFVTQRNFY
B10	LGVYYHKNNKSWMES	E2	LKPFERDISTEIYQA	G18	VFAQVKQIYKTPPIK	J10	HWFVTQRNFYEPQII
B11	HKNNKSWMESEFRVY	E3	RDISTEIYQAGSTPC	G19	KQIYKTPPIKDFGGF	J11	QRNFYEPQIITTDNT
B12	SWMESEFRVYSSANN	E4	EIYQAGSTPCNGVEG	G20	TPPIKDFGGFNFSQI	J12	EPQIITTDNTFVSGN
B13	EFRVYSSANNCTFEY	E5	GSTPCNGVEGFNCYF	G21	DFGGFNFSQILPDPS	J13	TTDNTFVSGNCDVVI
B14	SSANNCTFEYVSQPF	E6	NGVEGFNCYFPLQSY	G22	NFSQILPDPSKPSKR	J14	FVSGNCDVVIGIVNN
B15	CTFEYVSQPFLMDLE	E7	FNCYFPLQSYGFQPT	G23	LPDPSKPSKRSFIED	J15	CDVVIGIVNNTVYDP
B16	VSQPFLMDLEGKQGN	E8	PLQSYGFQPTNGVGY	G24	KPSKRSFIEDLLFNK	J16	GIVNNTVYDPLQPEL
B17	LMDLEGKQGNFKNLR	E9	GFQPTNGVGYQPYRV	H1	SFIEDLLFNKVTLAD	J17	TVYDPLQPELDSFKE
B18	GKQGNFKNLREFVFK	E10	NGVGYQPYRVVVLSF	H2	LLFNKVTLADAGFIK	J18	LQPELDSFKEELDKY
B19	FKNLREFVFKNIDGY	E11	QPYRVVVLSFELLHA	H3	VTLADAGFIKQYGDC	J19	DSFKEELDKYFKNHT
B20	EFVFKNIDGYFKIYS	E12	VVLSFELLHAPATVC	H4	AGFIKQYGDCLGDIA	J20	ELDKYFKNHTSPDVD
B21	NIDGYFKIYSKHTPI	E13	ELLHAPATVCGPKKS	H5	QYGDCLGDIAARDLI	J21	FKNHTSPDVDLGDIS
B22	FKIYSKHTPINLVRD	E14	PATVCGPKKSTNLVK	H6	LGDIAARDLICAQKF	J22	SPDVDLGDISGINAS
B23	KHTPINLVRDLPQGF	E15	GPKKSTNLVKNKCVN	H7	ARDLICAQKFNGLTV	J23	LGDISGINASVVNIQ
B24	NLVRDLPQGFSALEP	E16	TNLVKNKCVNFNFNG	H8	CAQKFNGLTVLPPLL	J24	GINASVVNIQKEIDR
C1	LPQGFSALEPLVDLP	E17	NKCVNFNFNGLTGTG	H9	NGLTVLPPLLTDEMI	K1	VVNIQKEIDRLNEVA
C2	SALEPLVDLPIGINI	E18	FNFNGLTGTGVLTES	H10	LPPLLTDEMIAQYTS	K2	KEIDRLNEVAKNLNE
C3	LVDLPIGINITRFQT	E19	LTGTGVLTESNKKFL	H11	TDEMIAQYTSALLAG	K3	LNEVAKNLNESLIDL
C4	IGINITRFQTLLALH	E20	VLTESNKKFLPFQQF	H12	AQYTSALLAGTITSG	K4	KNLNESLIDLQELGK
C5	TRFQTLLALHRSYLT	E21	NKKFLPFQQFGRDIA	H13	ALLAGTITSGWTFGA	K5	SLIDLQELGKYEQYI
C6	LLALHRSYLTPGDSS	E22	PFQQFGRDIADTTDA	H14	TITSGWTFGAGAALQ	K6	QELGKYEQYIKWPWY
C7	RSYLTPGDSSSGWTA	E23	GRDIADTTDAVRDPQ	H15	WTFGAGAALQIPFAM	K7	YEQYIKWPWYIWLGF

C8	PGDSSSGWTAGAAAY	E24	DTTDAVRDPQTLEIL	H16	GAALQIPFAMQMAYR	K8	KWPWYIWLGFIAGLI
C9	SGWTAGAAAYYVGYL	F1	VRDPQTLEILDITPC	H17	IPFAMQMAYRFNGIG	K9	IWLGFIAGLIAIVMV
C10	GAAAYYVGYLQPRTF	F2	TLEILDITPCSFGGV	H18	QMAYRFNGIGVTQNV	K10	IAGLIAIVMVTIMLC
C11	YVGYLQPRTFLLKYN	F3	DITPCSFGGVSVITP	H19	FNGIGVTQNVLYENQ	K11	AIVMVTIMLCCMTSC
C12	QPRTFLLKYNENGTI	F4	SFGGVSVITPGTNTS	H20	VTQNVLYENQKLIAN	K12	TIMLCCMTSCCSCLK
C13	LLKYNENGTITDAVD	F5	SVITPGTNTSNQVAV	H21	LYENQKLIANQFNSA	K13	CMTSCCSCLKGCCSC
C14	ENGTITDAVDCALDP	F6	GTNTSNQVAVLYQDV	H22	KLIANQFNSAIGKIQ	K14	CSCLKGCCSCGSCCK
C15	TDAVDCALDPLSETK	F7	NQVAVLYQDVNCTEV	H23	QFNSAIGKIQDSLSS	K15	GCCSCGSCCKFDEDD
C16	CALDPLSETKCTLKS	F8	LYQDVNCTEVPVAIH	H24	IGKIQDSLSSTASAL	K16	GSCCKFDEDDSEPVL
						K17	FDEDDSEPVLKGVKL
						K18	DDSEPVLKGVKLHYT

Table S3: List of the synthesized peptides covering the sequence of RBD mutated spike proteins from SARS-CoV-2 variants. Positive controls: YPGEFADYEELREQL (*Influenza virus),* spots 09 (A, B, C, D, E and F); negative controls: QEVRKY (*Cowpox vírus);* spot 11 (A, B, C, D, E and F); KEVPALTAVETGATN (Poliovirus) spot 10 (A, B, C, D, E and F).

Spot	Peptides	Spot	Peptides	Spot	Peptides
A1	GGGCVADYSVLYNGG	B1	GGGCVADYSVLYNGG	C1	GGGCVADYSVLYNGG
A2	GGGYADSFVIRGDGG	B2	GGGYADSFVIRGDGG	C2	GGGYADSFVIRGDGG
A3	GGGYNYKLPDDFTGG	B3	GGGYNYKLPDDFTGG	C3	GGGYNYKLPDDFTGG
A4	WNSNNLDSKVGGNYN	B4	WNSNNLDSKVGGNYN	C4	WNSNNLDSKVGGNYN
A5	GGGLKPFERDISTGG	B5	GGGLKPFERDISTGG	C5	GGGLKPFERDISTGG
A6	PLQSYGFQPTNGVGY	B6	PLQSYGFQPTNGVGY	C6	PLQSYGFQPTNGVGY
A7	PLRSYSFRPPYGVGH	B7	PLRSYSFRPPYGVGH	C7	PLRSYSFRPPYGVGH
A8	PLQSYGFRPTYGVGH	B8	PLQSYGFRPTYGVGH	C8	PLQSYGFRPTYGVGH
A9	YPGEFADYEELREQL	B9	YPGEFADYEELREQL	C9	YPGEFADYEELREQL
A10	KEVPALTAVETGATN	B10	KEVPALTAVETGATN	C10	KEVPALTAVETGATN
A11	GGGQEVRKYGGG	B11	GGGQEVRKYGGG	C11	GGGQEVRKYGGG
